# Model-Based Control of Soft Actuators Using Learned Non-linear Discrete-Time Models

**DOI:** 10.3389/frobt.2019.00022

**Published:** 2019-04-09

**Authors:** Phillip Hyatt, David Wingate, Marc D. Killpack

**Affiliations:** ^1^Robotics and Dynamics Lab, Department of Mechanical Engineering, Brigham Young University, Provo, UT, United States; ^2^Perception, Control, Cognition Lab, Department of Computer Science, Brigham Young University, Provo, UT, United States

**Keywords:** soft robot control, soft robot actuation, model predictive control, DNN, machine learning

## Abstract

Soft robots have the potential to significantly change the way that robots interact with the environment and with humans. However, accurately modeling soft robot and soft actuator dynamics in order to perform model-based control can be extremely difficult. Deep neural networks are a powerful tool for modeling systems with complex dynamics such as the pneumatic, continuum joint, six degree-of-freedom robot shown in this paper. Unfortunately it is also difficult to apply standard model-based control techniques using a neural net. In this work, we show that the gradients used within a neural net to relate system states and inputs to outputs can be used to formulate a linearized discrete state space representation of the system. Using the state space representation, model predictive control (MPC) was developed with a six degree of freedom pneumatic robot with compliant plastic joints and rigid links. Using this neural net model, we were able to achieve an average steady state error across all joints of approximately 1 and 2° with and without integral control respectively. We also implemented a first-principles based model for MPC and the learned model performed better in terms of steady state error, rise time, and overshoot. Overall, our results show the potential of combining empirical modeling approaches with model-based control for soft robots and soft actuators.

## 1. Introduction

**Figure 1:**
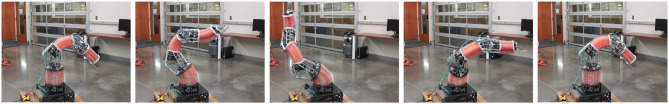
Series of joint configurations showing how this six degree of freedom pneumatic continuum robot can move.

Although model-based control can generally result in control that is superior to methods that do not rely on models, it is often difficult to justify the effort required to perform system identification or model development for complex systems. A common result is that we identify models that describe the system dynamics poorly and result in control that is barely (if at all) on par with basic feedback control methods such as PID control. As an example, soft robots are especially hard to model accurately for model-based control. The specific platform shown in Figure 1 has three joints that are made of antagonistic blow-molded plastic pneumatic chambers, where each joint has two degrees of freedom. In past work, Gillespie et al. (2018), we have shown that for a single degree of freedom soft robot, we could learn a model that performed on par with a linear model that we derived from first principles and traditional system identification techniques. However, in the case of the platform in Figure 1, we have all of the same problems that exist with the previous one degree of freedom platform (e.g., non-linear gas dynamics, hysteresis in joint behavior, state dependent stiffness and torque output, etc.), in addition to having to deal with linearizing and discretizing a 24 dimensional non-linear set of ordinary differential equations to describe the rigid body dynamics of a 3-link, 6-DoF pneumatic robot. As a first step toward learning models for soft robot control with a large number of degrees of freedom, we show that we can learn a discretized, non-linear model of the full robot from a non-linear simulation which allows us to achieve better control performance than the linearized model that is based on first principles. Although we are not advocating ignoring all physical intuition, we propose in this paper that it is possible to use recent advances in machine learning to rapidly develop an empirical model that can handle some of the non-linearities and complexities listed above for this system, and that can be used for control.

To model the unknown dynamics of our soft robot, we turn to the tools of deep learning. Deep learning is one of the most compelling advances in machine learning in recent memory. It has swept over both industry and academia, crushing benchmarks and generating impressive progress across fields as diverse as speech recognition (Dahl et al., 2012; Hinton et al., 2012; Deng et al., 2013; Abdel-Hamid et al., 2014), parsing of natural scenes (Lee et al., 2009; Socher et al., 2011), machine translation (Cho et al., 2014; Sutskever et al., 2014; Zhang and Zong, 2015), robotics (Eitel et al., 2015; Wulfmeier et al., 2015; Zhang et al., 2015; Levine et al., 2016), machine vision (Krizhevsky et al., 2012; Schmidhuber, 2012; Szegedy et al., 2015; Zeng et al., 2015), and even the game of Go (Silver et al., 2016).

A system like the robot described in this work, with severe hysteresis and unknown state interactions, is difficult to model even with explicit non-linear dynamics. These difficult-to-model dynamics are a perfect candidate for universal function approximation with deep neural networks, or DNNs. The only requirements for the approach proposed in this paper are that we must define the state variables and inputs based on our physical intuition about the problem. Additionally, we must be able to record data at each time step for our current states and randomized control inputs. Then we can train a deep neural net to approximate the non-linear, discretized dynamics and then linearize that model at each time step for control.

Our specific contributions include the following:

- Development of a non-linear neural network (NN) architecture for dynamic modeling of a 6 DoF pneumatic robot with soft actuators based on data from a full non-linear model.
- Development of a model predictive controller that can use the partial derivatives of the NN at every time step in order to remain tractable for low-level, high-bandwidth control while modeling joint configurations, joint velocities, and joint pressures.
- Development of a first-principles-based model and a model predictive controller for a 6 DoF continuum pneumatic robot with soft actuators.
- Identification of specific open questions relative to learning more accurate dynamic models for future model-based soft robot control.
- Validation and benchmarking of the non-linear NN model for model-based control against the first principles model for a large number of degrees of freedom.

The last contribution is especially interesting as we expect this approach to generalize to other difficult-to-model actuators or robot systems where model-based control would be expected to improve low-level control performance but system identification or even model development is particularly difficult.

The rest of this paper is organized as follows, we first describe related work in section 2.1. In section 2.2, we describe our robot platform. Section 2.3 describes the modeling and control of the robot. Our results are presented in section 3 and we discuss the results in section 4.

## 2. Materials and Methods

### 2.1. Related Work

Past research that is related to the work we present in this paper can be divided into two main areas. The first is using neural networks either as a model for model-based control or as a controller itself. The second area is other parametric models that are used to produce optimal control policies. After discussing these areas, we also briefly address related work on controlling soft robots. More background on research using model predictive control in robotics can be found in Jain (2013) and Best et al. (2016). However, it is important to note that model predictive control solves a finite horizon optimal control problem at each time step subject to the model dynamics as an equality constraint along with any other defined constraints on the states and inputs.

#### 2.1.1. Other Learned Models for Model-Based Control

Although traditional robotics modeling has focused on system identification of traditional physics-based models (see Swevers et al., 1997; Park et al., 2011), the last 20 years has seen a significant increase in the number of empirical models and methods that have been developed (see Nguyen-Tuong and Peters, 2011). One common approach is to use Gaussian Processes (GP) to model the dynamic system and this seems to have first been done in the chemical processing industry (see Kocijan et al., 2004 for example). Currently in robotics, GP has been used to develop a policy search algorithm for a robot arm with imprecise actuators and cheap sensors (Deisenroth et al., 2011), or for more general purpose control policy development (Deisenroth et al., 2015). Other researchers have used Gaussian Mixture Models such as in Calinon et al. (2007). We make no direct comparison to these other modeling methods in this paper, but expect that this would be a worthwhile comparison in future work.

Although there exists a large number of other learning methods that we could have used or compared against (e.g., Gaussian Processes, or support vector machines), we have chosen DNNs for their unique properties that make them an ideal choice for this application. Specifically, both GPs and SVMs require fixed-size data sets, but our vision is to extend this work to on-line scenarios where system identification happens concurrently with control. In addition, an important aspect of using DNNs is differentiability. Part of the appeal of using DNNs is the fact that many off-the-shelf frameworks for deep learning (Tensorflow, MXNet, pytorch) all support automatic differentiation. This makes it easy to compute the gradients of dynamics with respect to control inputs, which is needed for MPC. In contrast, we are not aware of any existing GP/SVM packages that have similar capabilities.

#### 2.1.2. Neural Nets

The approach of using neural networks (similarly to model predictive control), appears to have come from the chemical processing industry and work that is most relevant to our approach for robotics was found in Psichogios and Ungar (1991), Piche et al. (1999), and Draeger et al. (1995) and is still an active area of research (Patan, 2015).

Early work using neural networks for modeling robots was done in Kiguchi et al. (1999), but it was not used for control. In Tan et al. (2007) they use neural networks to learn disturbance models online while controlling, while in Huang et al. (2000) and Huang et al. (2002) radial basis functions are used to learn friction effects modeled in an adaptive control scheme. These adaptive control ideas could be particularly applicable to our platform in future work as the air bladders in our soft robot tend to wear or shift over time, which then changes the dynamic model. In Yan and Wang (2011) Yan and Wang use a recursive neural net to represent the higher order error terms that result from a Taylor series linearization. While in Yan and Wang (2014) they use a minimax optimization and learn a neural net model for part of their unknown dynamics. In both cases, the only results shown are in simulation.

More recent work has focused on learning controllers or models for high-level tasks. In Lenz et al. (2015) for example, they use a recurrent neural net to learn features of specific classes of fruits and other foods in order to more efficiently slice the food. The formulation is application specific but uses the neural network gradients similarly to our approach. For the work in Zhang et al. (2016), they used a DNN to learn a MPC control policy for UAVs. Levine and Abbeel do policy search using locally linear dynamics models to learn neural network controllers for different robot tasks (e.g., swimming, insertion) in Levine and Abbeel (2014). Finally, Fu et al. (2015) use neural networks to generate and adapt models online that can be used for model-based reinforcement learning to learn a control policy that makes use of iterative LQR. Although the output is a low-level torque for each joint, this approach does not generalize to more basic capabilities such as force or position control. Low-level control is our current interest given the inherent non-linearity and complexity of our platform even without interacting with complicated environments.

In general, using neural nets for system identification is a well-known method (Narendra and Parthasarathy, 1990), but the novelty of our paper is that we apply the method to control soft robot platforms by combining neural nets with MPC for low-level control. There is an inherent trade-off for controllers when using these types of black box modeling. The trade-off is that we can either develop controllers that can be used for multiple tasks when the tasks can be decomposed into specific and explicit force or position requirements or we can develop controllers for tasks where even the desired force and position profiles are uncertain with respect to the robot hardware. In the second case, learning the task rather than (or in addition to) the robot dynamics is necessary but is a next step to the work we present here for low-level control of soft robots.

#### 2.1.3. Soft Robot Control

A significant portion of soft robot research described in the survey (Rus and Tolley, 2015) was focused on design methodologies instead of closed-loop control performance and so most robots were controlled with open-loop strategies such as in Shepherd et al. (2011) and Tolley et al. (2014). Research that is most related to ours in terms of trying to control a robot to a specific configuration includes the use of inflatable links with cable tendons (Sanan et al., 2009, 2011), fluid drive elastomer (Marchese et al., 2014a,b; Marchese and Rus, 2015), or rotary elastic chamber actuators such as in Ivlev (2009) and Gaiser et al. (2014). However, in addition to other differences with past soft robot control work that we outline more specifically in Best et al. (2016), as far as we know they have not developed control for these robots based on learned empirical models like those that we present in this paper. This is true except for work in Thuruthel et al. (2017) where they learn dynamic models similar to what we present. However, they use those models in an open-loop control scheme which may be problematic in the case of model error, change over time, or any kind of disturbance. A recent survey that explains the state of the field for soft and continuum robot control can be found in George Thuruthel et al. (2018).

### 2.2. Robot Platform Description

**Figure 2:**
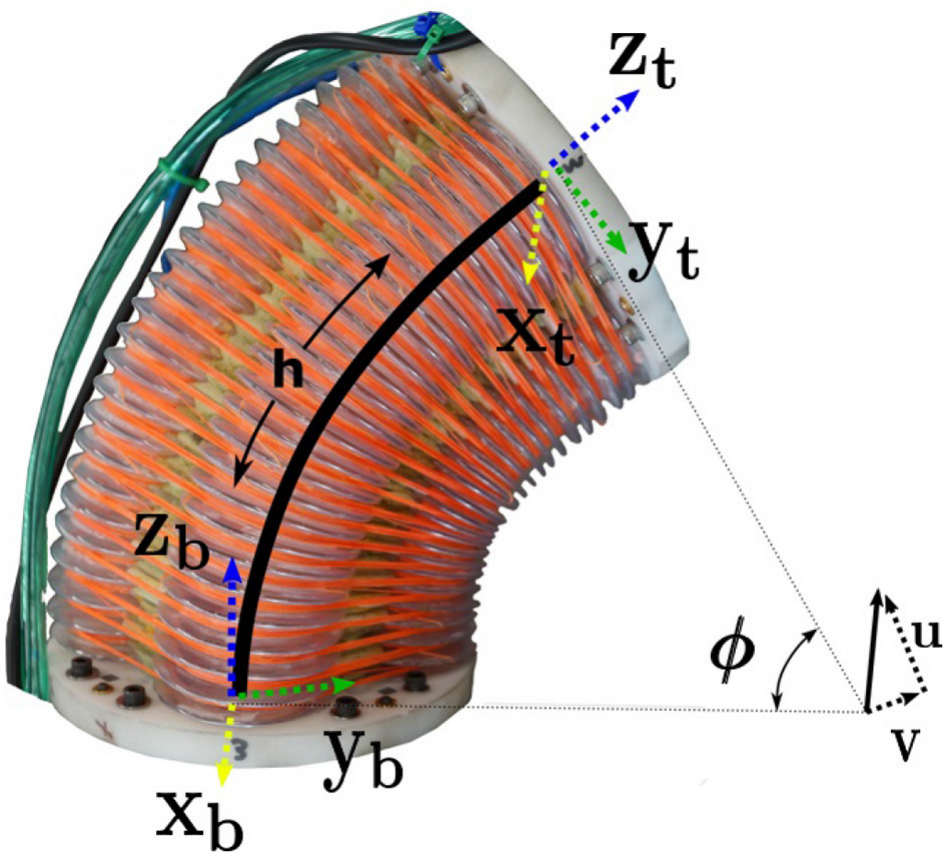
Kinematic frames and model used for blow-molded continuum joint.

The platform used for this research was a compliant, continuum robot with six actuated degrees of freedom (see Figure 1). Each joint consists of four pneumatic chambers made of blow-molded plastic. The top and bottom sections of the actuator are connected with a cable putting the bellow actuators in compression to some degree. By filling or venting each chamber, we can get motion about two different axes of rotation as shown in Figure 2. The platform was developed and built by Pneubotics, an affiliate of Otherlab. In our previous work, we applied the methods presented in this paper on a one degree of freedom, fabric-based, soft robot joint. In this paper, the platform we use still has soft joints and is pneumatic. However, rigid components are used to connect the soft joints. The motivation for using learning is however unchanged since the joints exhibit very non-linear behavior including the gas dynamics, hysteresis, non-linear stiffness, and other non-linearities.

Because each joint is a continuum with two degrees of freedom, it is necessary to describe its configuration with two joint variables. We represent rotation at the joint using a vector constrained to be in the plane of the base of the joint as seen in Figure 2. Because it is constrained to a plane, the vector only has two components which we name *u* and *v*. These are our joint configuration variables. The magnitude of this vector (ϕ) is the rotation angle about a single axis, while the vector describes that rotation axis. Joint configuration estimation is accomplished in real-time using software supplied by the manufacturer. This software uses data from IMUs mounted on the rigid links to estimate *u*, *v*, $\dot{u}$, and $\dot{v}$.

Joints are actuated by controlling pressure separately in each of the 4 chambers in each joint (12 pressures total for the arm). While the bottom joint has 8 chambers, these are controlled in pairs of two, so it is effectively a larger version of the 4 chamber joints. For this work, supply pressure was maintained at 70 PSI while pressures in the chambers were limited between 18 and 55 PSI by the controller. These pressure limits were enforced to ensure the robot did not damage itself.

We use the Robot Operation System (ROS) to access state estimates as well as to send pressure commands. Our MPC controller code is operating in non-realtime on an Ubuntu workstation, while the state estimation and low level pressure control is being executed at 1,000 Hz on a PC with a real-time linux kernel.

### 2.3. Development of Dynamic Models

#### 2.3.1. First Principles Dynamics Model

A model of the evolution of system states was derived from first principles based on material properties, lengths, and masses provided by the manufacturer of the robot. Because commanded pressures were not achieved instantaneously, it was deemed necessary to model the dynamics of pressures and the high rate pressure controller. The entire state of our system is therefore $\text{x}={\left[p,\dot{q},q\right]}^{T}$ where *p* is the vector of the pressures in the 12 chambers (4 per joint), $\dot{q}$ is the vector of 6 joint velocities ($\left[{\dot{u}}_{1},{\dot{v}}_{1},{\dot{u}}_{2},{\dot{v}}_{2},{\dot{u}}_{3},{\dot{v}}_{3}\right]$), and *q* is the vector of 6 joint positions ([*u*_1_, *v*_1_, *u*_2_, *v*_2_, *u*_3_, *v*_3_]). The inputs to our system are **u** = [*p*_*ref*_] where *p*_*ref*_ is a column vector of commanded pressures sent to the high rate PID pressure controller.

The pressure dynamics were modeled as first order according to the differential equation

(1) $$\begin{array}{l} ṗ=\alpha \left(p_{ref}-p\right) \end{array}$$

where α is a diagonal matrix of constant coefficients which represent the speed of filling or venting a chamber.

The dynamics of the links were modeled using the equation

(2) $$\begin{array}{l} M\left(q\right)\ddot{q}+C\left(\dot{q},q\right)\dot{q}=K_{d}\dot{q}+K_{spring}q+\tau_{grav}+K_{prs}p \end{array}$$

where *M*(*q*) is the joint space inertia matrix, $C\left(\dot{q},q\right)$ is the joint space Coriolis matrix, *K*_*spring*_ and *K*_*d*_ are spring and viscous damping terms which are significant in our elastic continuum joints, τ_*grav*_ is a vector of the torques caused by gravity, *K*_*prs*_ is a matrix which maps the pressures in the chambers to torques at the joints. While this model could benefit greatly from further system identification, we report results using this model and leave model improvement for future work.

Placing all of the state variables and derivatives into state space form we can write

(3) $$\dot{\text{x}}=Ax+Bu+w$$

where

(4) $$\begin{array}{l} \text{A}=\left[\begin{array}{ccc} -\alpha & 0 & 0\\ M^{-1}K_{prs} & M^{-1}\left(K_{d}-C\right) & M^{-1}K_{spring}\\ 0 & 1 & 0 \end{array}\right] \end{array}$$

(5) $$\begin{array}{l} \text{B}=\left[\begin{array}{c} \alpha \\ 0\\ 0 \end{array}\right] \end{array}$$

(6) $$\begin{array}{l} \text{w}=\left[\begin{array}{c} 0\\ \tau_{grav}\\ 0 \end{array}\right] \end{array}$$

By writing the model in this way, we are assuming that the state dependent matrices in *A* change slowly over the time horizon in our controller [similar to our models in past work Killpack et al. (2016) for rigid robots with compliance at the joints]. The discretization of the continuous time state space matrices is done using the matrix exponential, which gives

(7) $$x_{k+1}=A_{d}x_{k}+B_{d}u+w_{d}.$$

where

(8) $$\begin{array}{l} {\text{A}}_{d}=e^{\text{A}\Delta t} \end{array}$$

(9) $$\begin{array}{l} {\text{B}}_{d}={\text{A}}^{-1}\left({\text{A}}_{d}-\text{I}\right)\text{B} \end{array}$$

(10) $$\begin{array}{l} {\text{w}}_{d}=\text{w}\Delta t \end{array}$$

#### 2.3.2. Deep Neural Net Architecture and Model

**Figure 3:**
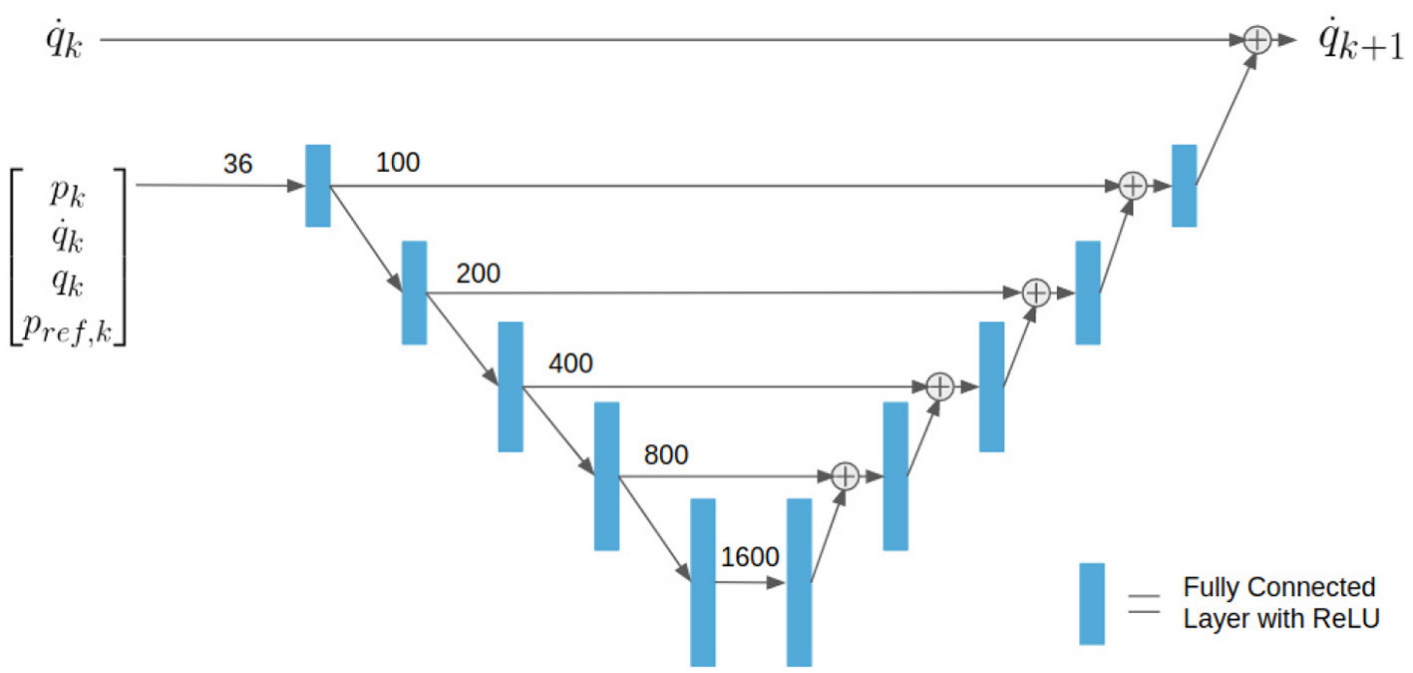
DNN architecture implemented to predict future velocities.

A Deep Neural Net (DNN) of the form shown in Figure 3 was trained as a discrete-time dynamic model for the velocity states of our system. Because we have fairly accurate and simple representations for pressure and position, we represent those using first principles methods. The entire model consisted of about 3.4 million nodes in an architecture similar to the Unet architecture used for image processing (Ronneberger et al., 2015), except our architecture uses fully connected layers with ReLU activations instead of convolutions. The model used for this work was trained for less than 1 h on a NVIDIA Titan X GPU. The DNN can be described as finding the change in velocity between time steps *k* and *k*+1 taking as inputs the entire state and inputs at time *k*. Assuming our system is a first order Markov system, this approach should be very reasonable. The DNN can be represented as a function of the form

(11) $$\begin{array}{l} {\dot{q}}_{k+1}=f\left({\text{x}}_{k},{\text{u}}_{k}\right). \end{array}$$

As a method of collecting a large amount of data very quickly and without wear or danger to the actual robot, we used the non-linear first principles model outlined above (before discretization and linearization) to train the DNN. Non-linear simulation was accomplished by integrating the state space equations at a discretization of 0.001 s. In order to train the DNN to predict ${\dot{q}}_{k+1}$ at a discretization of 0.05 s, the non-linear simulation was carried out for 50 integration steps. Because *p*_*k*_, *q*_*k*_, and *p*_*ref*_ all have definite bounds, these were sampled uniformly within their bounds. However, $\dot{q_{k}}$ is not bounded, so samples were drawn from a mean zero normal distribution. In an attempt to scale the input space equally, *p* and *p*_*ref*_ were scaled and offset to be mean zero values between –1 and 1. Using units of radians *q* was bounded by +- $\frac{2\pi }{3}$.

**Figure 4:**
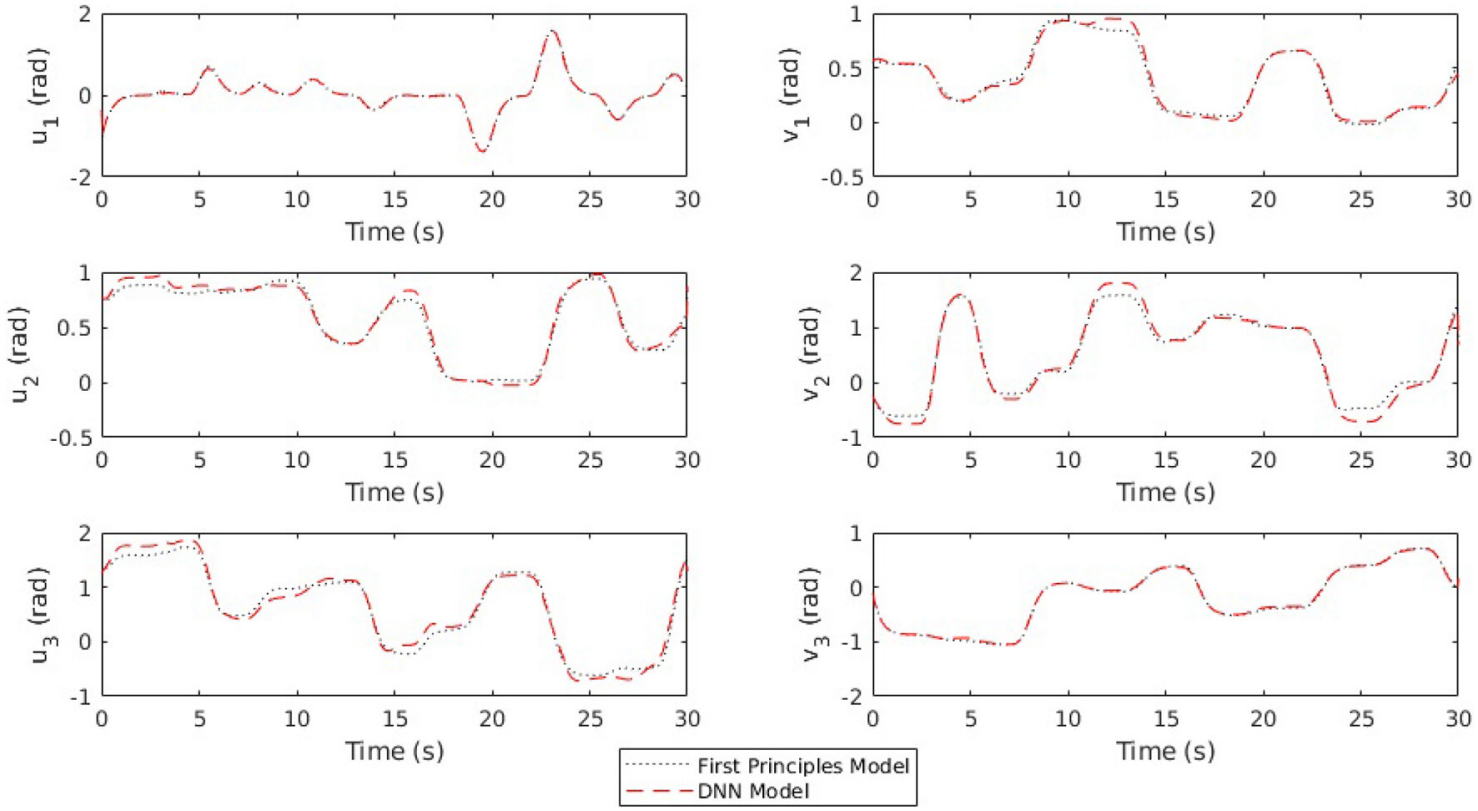
Simulations using the same initial states and a 30 s input trajectory are rolled out using the DNN model and the first principles model. For most of the trajectory, these lines are indistinguishable.

It should be noted that a method to learn new features while maintaining old ones could be used to improve this model (Rusu et al., 2016), however our control results demonstrated acceptable performance without this step. An example of open loop prediction of joint positions using the DNN compared to the first principles model and measured data can be seen in Figure 4. The error statistics for both position and velocity are reported in Table 1.

**Table 1:** Error statistics for a 30 s rollout of arm dynamics.

**Non-linear DNN prediction error**			
	**Max**	**Mean**	**Std. Dev**.
Velocity error (rad/s)	1.0721	−0.000003	0.0237
Position error (rad)	0.3736	−0.0012	0.0223

*Error is reported as the difference between the non-linear DNN model prediction and the non-linear simulation used to train the arm*.

Using a non-linear optimization, this non-linear model could be used for MPC, however in order to ensure that we solve at fast enough rates for real-time control we choose to linearize this DNN model using the Taylor Series expansion. The Taylor expansion of our DNN model (Equation 11) linearized about **x**_0_, **u**_0_ is

(12) $${\dot{q}}_{k+1}=\frac{\partial f}{\partial x_{k}}{|}_{x_{0},u_{0}}(x_{k}-x_{0})+\frac{\partial f}{\partial u_{k}}{|}_{x_{0},u_{0}}(u_{k}-u_{0})+f(x_{0},u_{0})$$

where the partial derivatives are of the DNN's outputs with respect to its inputs. While these partial derivatives of the entire non-linear DNN may be too long and complex to write by hand, they are easily obtained using the automatic differentiation library already included as part of the DNN training library.

Because the DNN only predicts the velocities at the next time step (${\dot{q}}_{k+1}$), we must supply a discrete model for pressures (*p*_*k*+1_) and positions (*q*_*k*+1_). For positions we use a simple numerical integration using the trapezoidal rule:

(13) $$\begin{array}{l} q_{k+1}=q_{k}+\frac{\Delta t}{2}\left({\dot{q}}_{k}+{\dot{q}}_{k+1}\right) \end{array}$$

while for pressures we use the simple discretization of Equation 1

(14) $$\begin{array}{l} p_{k+1}=\alpha \Delta t\left(p_{ref}\right)+\left(I-\alpha \Delta t\right)p_{k} \end{array}$$

The discrete-time state space equation for this system is given by

(15) $$x_{k+1}=A_{d}(x_{k}-x_{k})+B_{d}(u_{k}-u_{k})+w_{d}$$

where

(16) $$\begin{array}{l} {\text{A}}_{d}=\left[\begin{array}{ccc} \left(I-\alpha \Delta t\right) & 0 & 0\\ \frac{\partial f}{\partial p_{k}} & \frac{\partial f}{\partial {\dot{q}}_{k}} & \frac{\partial f}{\partial q_{k}}\\ \frac{\partial f}{\partial p_{k}}\frac{\Delta t}{2} & \left(\frac{\partial f}{\partial {\dot{q}}_{k}}+I\right)\frac{\Delta t}{2} & \frac{\partial f}{\partial q_{k}}\frac{\Delta t}{2}+I \end{array}\right] \end{array}$$

(17) $$\begin{array}{l} {\text{B}}_{d}=\left[\begin{array}{c} \alpha \Delta t\\ \frac{\partial f}{\partial p_{ref,k}}\\ 0 \end{array}\right] \end{array}$$

(18) $$\begin{array}{l} {\text{w}}_{d}=\left[\begin{array}{c} p_{0}\\ f\left({\text{x}}_{0},{\text{u}}_{0}\right)\\ \frac{\Delta t}{2}{\dot{q}}_{0}+q_{0} \end{array}\right] \end{array}$$

### 2.4. Model Predictive Control Development

**Figure 5:**
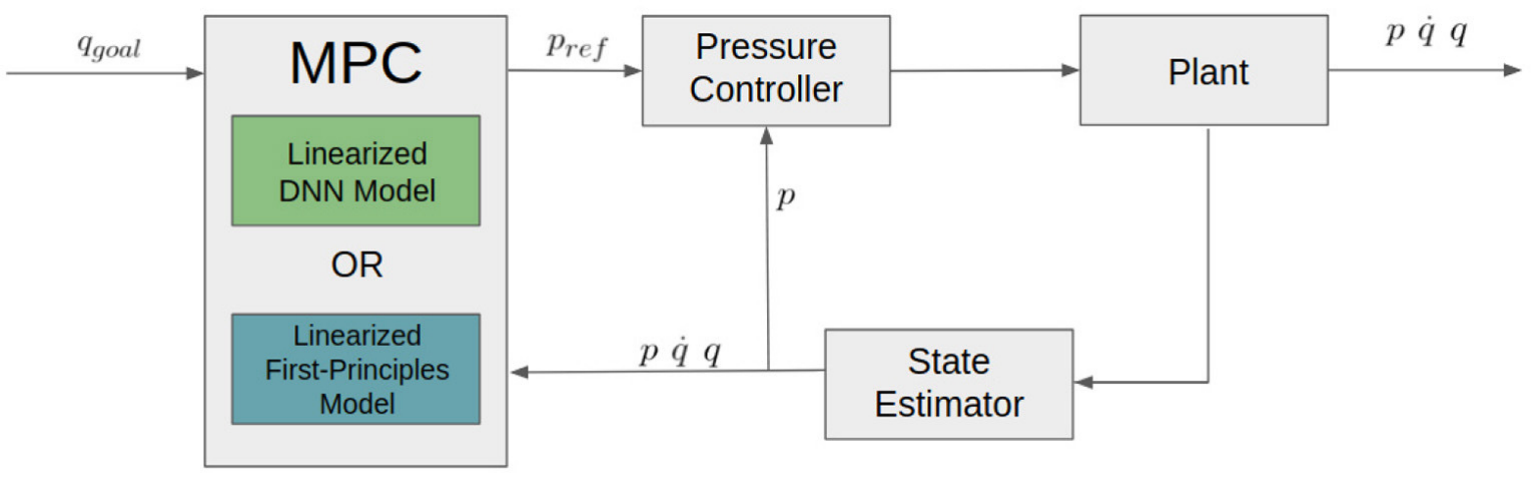
Control loop diagram showing the MPC controller sending pressure commands (p_ref_) to a low level PID pressure controller.

The linear discrete-time state space models (Equations 7, 15) are used as constraints in an MPC controller that is run at 20 Hz. A flow chart for the control process can be seen in Figure 5. The outputs from the model predictive controller are reference pressures that are sent to a low level PID pressure controller running at 1,000 Hz.

Feedback for the MPC controller is given by a state estimator supplied by the manufacturer. This state estimator uses IMUs and pressure sensors mounted on the arm to estimate *p*, $\dot{q}$, and *q*. This data is updated at a rate of 1,000 Hz.

The solver that we used for MPC was generated using CVXGEN (see Mattingley and Boyd, 2012 for more details about the optimization and constraint handling), a web-based tool for developing convex optimization solvers, with a horizon of 4 time steps. The cost function minimized across the horizon *T* is

(19) $$\begin{array}{l} minimize\overset{T}{\underset{k=1}{\sum }}∥q_{goal}-q_{k}{∥}_{Q}^{2}+∥p_{ref,k}-p_{ref,k-1}{∥}_{R}^{2} \end{array}$$

subject to the system model as constraints, as defined in Equations (7, 15) (the first principles and DNN dynamic models respectively), as well as the following additional constraints:

(20) $$\begin{array}{lll} q_{min} & \leq & q_{k}\leq q_{max}\forall k \end{array}$$

(21) $$\begin{array}{lll} p_{min} & \leq & p_{ref,k}\leq p_{max}\forall k \end{array}$$

where *Q* and *R* are scalar weights manually tuned for performance, *q*_*min*_ and *q*_*max*_ are the joint limits, and *p*_*min*_ and *p*_*max*_ are minimum and maximum pressures. It is important to note that the weights in the cost function for MPC are what will determine the performance of the control to a large degree in terms of traditional metrics like rise time, steady state error, and overshoot. Also note that the weighting matrix *R* penalizes change in pressure from one step to another. This term discourages very fast motions and eliminates the need for velocity constraints.

We used the exact same weights and constraints for all controller comparisons. We instead varied only the model (either based on first principles or the DNN learned model) and whether or not an integrator was used. When an integrator was used, the following equation was used to handle steady state error with integral action:

(22) $$\begin{array}{l} q_{goal,k+1}=q_{goal,k}+k_{i}\left(q_{goal,k}-q_{k}\right) \end{array}$$

When the integrator was used, it was only ever active when the combined error in joint angles versus their commanded angles was less than 0.4 radians to help with overshoot. Although step inputs are notorious for exciting overshoot and oscillation in underdamped systems, as opposed to trajectories smoothed with sinusoids or polynomials, we wanted to test the performance of our models and controllers and therefore we sent direct step inputs to each joint. In the future, these same commands could be smoothed to likely achieve better performance. However, the same could be argued if our models continue to improve and our model predictive controllers are able to make use of methods to predict further into the future (see more discussion in section 4).

## 3. Results

**Figure 6:**
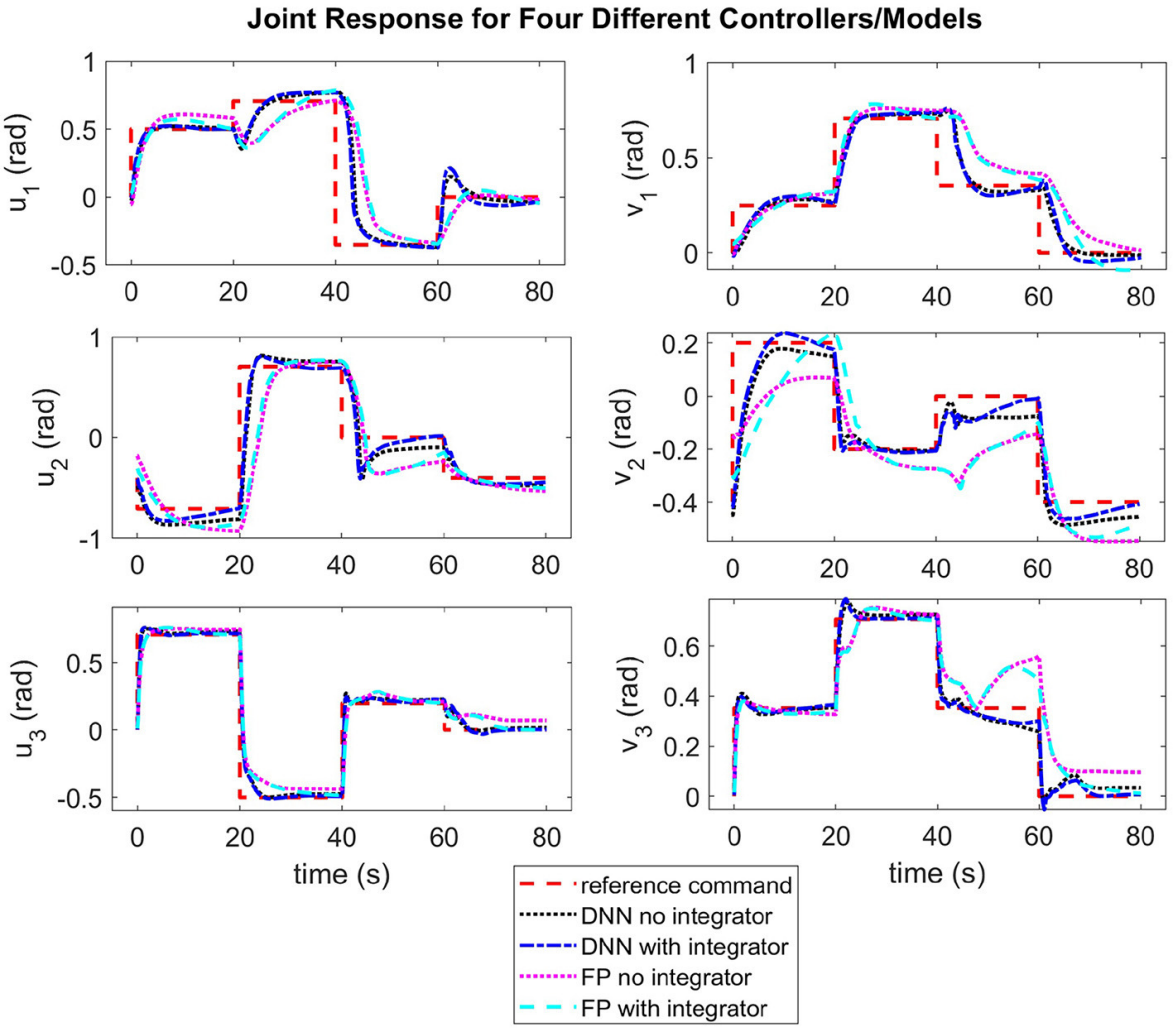
Each plot shows a comparison of different time responses for a single joint in a single direction of actuation (where each joint has a degree of freedom in the *u* and *v* directions as shown in Figure 2). For each joint, the time response for four different conditions is shown–(1)MPC with a deep neural net (DNN) model, (2)MPC with a DNN model and an integrator, (3)MPC with a first principles (FP) model, (4)MPC with a first principles (FP) model and integrator.

In each trial for our experiments, the same set of commanded joint angles (*u* and *v* for each joint) were sent to the controller with 20 s intervals between commands. The commanded angles are found in Table 2 and were selected in order to force the arm to move through most of its workspace. Step commands are not traditionally used in robotics due to the fact that they can induce unwanted dynamics or oscillation, even in traditional rigid robots. However, in this case, we want to test our controller's ability to use the model to mitigate unwanted behavior, similar to some of our past work (see Rupert et al., 2015; Terry et al., 2017). The results for the same model predictive controller using the two different linearized models (first-principles and DNN), and with and without integral action, are found in Figure 6. A video showing the robot moving through the same joint configurations as those found in Table 2 and shown in Figure 1 can be seen at https://youtu.be/ddA0g0yKjOc. The controller used in the video is the DNN MPC controller with no integral action. The video also shows the compliance of the soft actuators when perturbed by an external disturbance.

**Table 2:** This set of joint angle commands move the continuum robot arm throughout the workspace and are used to evaluate performance.

	**Joint angle commands**				
	Initial	Step 1	Step 2	Step 3	Final
*u*_1_	0.0	0.5	0.707	−0.354	0.0
*v*_1_	0.0	0.25	0.707	0.354	0.0
*u*_2_	−0.4	−0.707	0.707	0.0	−0.4
*v*_2_	−0.4	0.2	−0.2	0.0	−0.4
*u*_3_	0.0	0.707	−0.5	0.2	0.0
*v*_3_	0.0	0.354	0.707	0.354	0.0

In addition, the median steady state error, rise time, and overshoot for each joint and each controller is included in Table 3. We also included an average across all the joints for each controller and the controller that performed the best is shown in bold. Where C1 refers to the first principles MPC without integral control, C2 refers to the first principles MPC with integral control, C3 refers to the DNN MPC without integral control, and C4 refers to DNN MPC with integral control. In all cases, the controller that performed the best was one of the neural network model predictive controllers (C3 and C4, without or with integral control).

**Table 3:** The average steady state error, 90% rise time, and percent overshoot are all reported for each joint and controller.

	**Steady state error (rad)**					**(10–90%) Rise time (s)**					**% Overshoot**			
	**C1**	**C2**	**C3**	**C4**		**C1**	**C2**	**C3**	**C4**		**C1**	**C2**	**C3**	**C4**
*u*_1_	0.029	0.032	0.030	0.030		5.04	4.38	2.38	2.19		5.50	6.29	4.48	4.91
*v*_1_	0.046	0.046	0.020	0.020		10.2	7.22	3.60	3.41		36.4	35.8	32.1	34.9
*u*_2_	0.157	0.112	0.079	0.019		3.77	3.83	2.21	2.11		17.5	14.7	13.9	11.5
*v*_2_	0.124	0.074	0.047	0.012		11.5	9.94	7.00	5.68		18.7	22.8	6.11	10.4
*u*_3_	0.048	0.006	0.024	0.010		6.61	4.19	1.77	1.82		12.1	12.8	11.2	8.22
*v*_3_	0.086	0.033	0.038	0.019		7.61	4.84	0.71	0.55		29.8	28.1	33.0	31.3
Total Average	0.0816	0.051	0.040	**0.018**		7.46	5.74	2.94	**2.63**		20.0	20.1	**16.8**	16.9

*In cases where 90% of steady state was not reached, a full 20 s was counted for that step. In cases where there was not overshoot, it was counted as 0% for that step*.

Although it may be desirable to track sinusoids or other trajectories for different applications, step inputs are the most difficult input for underdamped systems. In this case, if the soft, underdamped robot gives good control performance without having to slew the control input, we have evidence that the learned model is effective.

## 4. Discussion of Results, Future Work, and Conclusions

One of the most interesting results is that the both models make full use of the multi-input system by driving pressures in opposing chambers in opposite directions in order to get a joint to move more quickly. This is something that we expect to see in the first principles when we explicitly model torque as a function of the two actuation pressures. However, in the DNN model, the behavior of the system was learned automatically by the DNN and exploited by the model predictive controller.

Both approaches have strengths and weaknesses with respect to ease of implementation. Given decent model parameters, a first principles model can be derived and verified with real data. This allows you to see the predictive power of your model and to reason about where errors are being introduced (e.g., underestimating mass causes velocities to be higher). While training a DNN model on data is theoretically much simpler and requires less system and theoretical knowledge, in practice it can be difficult to obtain large quantities of high quality data with which to train, especially on real robots. Moreover, if the DNN model does not predict well, it is difficult to discern if the problem is with the architecture, the training method, the data, or simply the quantity of the data.

Once both methods are implemented, it is again theoretically much simpler to update the DNN model given new data. This could be useful for slow system changes due to phenomenon such as creep or possibly even for quick system changes such as when an object is grasped in a robotic end effector. The equivalent process with a first principles model is adaptive control, which is still an active area of research. Whether one of these is simpler in practice remains for exploration in future work.

As can be seen in Figure 6 and Table 3, the DNN MPC was able to control to positions with lower rise time, overshoot, and steady state error, than the first principles MPC. This is interesting especially because the DNN was trained exclusively on data produced by the non-linear first principles model. We expect that this performance increase is in part due to how the DNN model is linearized (using the Taylor series expansion) as opposed to how the first principles model is linearized (maintaining **A** and **B** constant). This is supported in the findings of Terry et al. (2017). However, this ends up being one of the benefits of the learned model. It handles, the discretization *and* linearization in a more straight forward way than the when dealing with the non-linear first principles model, while still giving comparable or better performance.

It should be noted that in the DNN model, only velocities were predicted using the DNN, while pressures and positions were found using first principles models and discretization techniques. This was done because it proved to be much more difficult to train a DNN capable of predicting the entire state vector, as opposed to just velocities. While predicting the entire state was successfully accomplished in Gillespie et al. (2018), this was for a one degree of freedom system. We suspect that in order to extend this directly to many degrees of freedom, the DNN model would need to be much larger and be trained on much more data, or the DNN architecture would need to be changed to constrain the model to be more physically realistic. Since larger models require more data and would require more time to calculate gradients for control, smaller DNNs can be more useful in practice. We pose to the community as an open problem the correct architecture for discrete-time model prediction of dynamic states, since this will have a great impact on model and controller performance as well as training time and the amount of data required. Another open question is how to most safely and effectively collect data for learning the dynamics of a system such as a real robot, without damaging the robot.

Additionally, it is important to keep in mind that in this paper we are using a simple first-order Markov, feed-forward NN which cannot capture hysteresis. However, in future work, our same approach could be applied with more advanced DNN versions that can model hysteresis and other similar physical phenomenon. For example, it is possible to use k-th order Markov inputs, or train DNNs with state (e.g., LSTMs or GRUs) to remember inflection points.

We have shown that using a DNN with no initial knowledge about a complicated non-linear dynamical system except for assumed state variables and inputs, we can develop a high-performing model-based controller. Additionally, we have shown that our method which was first presented in Gillespie et al. (2018) is extensible to a more complex and large degree of freedom robot with soft actuators. In preliminary testing, the model predictive controller using a learned model performed better in terms of both overshoot and steady state error than a model predictive controller using a simplified linearized model based on first principles. Despite this success, we also note that it will be important in future work to extend our methods in two main ways. First we expect that constraining or parameterizing the model appropriately to cause a learned model to predict better after being trained on real data (as opposed to a non-linear simulation) will be essential to further improving performance. Additionally, in order to further improve dynamic response (such as rise time, overshoot, and settling time) we expect that using more tractable MPC methods with longer horizons and higher control rates (such as the method in Hyatt and Killpack, 2017 which make use of a GPU) will allow us to better control underdamped, difficult to model, soft robot actuators.

## Author Contributions

PH and MK contributed to the controller and first principles model development. PH and DW contributed to the design and training of the neural net. PH and MK tuned the MPC with all models and collected the data reported in the paper. All authors contributed to manuscript revision, read, and approved the submitted version.

### Conflict of Interest Statement

The authors declare that the research was conducted in the absence of any commercial or financial relationships that could be construed as a potential conflict of interest.
